# COVID-19 Pandemic as an Excellent Opportunity for Global Health Diplomacy

**DOI:** 10.3389/fpubh.2021.655021

**Published:** 2021-07-12

**Authors:** Sanaz Taghizade, Vijay Kumar Chattu, Ebrahim Jaafaripooyan, Sebastian Kevany

**Affiliations:** ^1^Department of Health Management and Economics, School of Public Health, Tehran University of Medical Sciences, Tehran, Iran; ^2^Department of Medicine, Faculty of Medicine, University of Toronto, Toronto, ON, Canada; ^3^Institute of International Relations, The University of the West Indies, St. Augustine, Trinidad and Tobago; ^4^Daniel K. Inouye Asia-Pacific Center for Security Studies, Honolulu, HI, United States

**Keywords:** COVID-19, pandemic, foreign policy, global health diplomacy, international cooperation, health security, health systems, trade

## Abstract

Undoubtedly, the COVID-19 pandemic is not the first and most frightening global pandemic, and it may not be the last. At the very least, this phenomenon has though seriously challenged the health systems of the world; it has created a new perspective on the value of national, regional, and international cooperation during crises. The post-coronavirus world could be a world of intensified nationalist rivalries on the economic revival and political influence. However, strengthening cooperation among nations at different levels will lead to the growth of health, economy, and security. The current situation is a touchstone for international actors in coordinating the efforts in similar future crises. At present, this pandemic crisis cannot be resolved except through joint international cooperation, global cohesion, and multilateralism. This perspective concludes that the pandemic could be an excellent opportunity for the scope of global health diplomacy (GHD) and how it can be applied and practiced for strengthening five global arenas, namely (1) International Cooperation and Global Solidarity, (2) Global Economy, Trade and Development, (3) Global Health Security, (4) Strengthening health systems, and (5) Addressing inequities to achieve the global health targets. GHD proves to be very useful for negotiating better policies, stronger partnerships, and achieving international cooperation in this phase with many geopolitical shifts and nationalist mindset among many nations at this stage of COVID-19 vaccine roll-out.

## Introduction

The first case of COVID-19 reported in Wuhan, China, in December 2019, was declared as an international public health concern and a global pandemic by the World Health Organization (WHO) on March 11, 2020 (1). As of May 17, 2021, nearly 162.8 million are infected, and over 3.37 million deaths reported to WHO globally, with the death toll still mounting. As of May 17, 2021, a total of 1.26 billion vaccine doses have been admistered (2). Because the pandemic is also a threat to global health and economy and given our relatively limited knowledge of treatment and prevention options, countries should cooperate and maintain the dialogue. The nations need to continue to share their experiences, successful policies, and measures in addition to the necessary information to successfully control the disease and reduce its devastating public health, economic, and social consequences. As stated by the WHO Director-General: “the first window of opportunity to prevent the disease spread to other countries during the past 1 to 2 months is missed. Hence, countries should do their best to avoid missing the second window” (1). Viruses such as SARS-CoV-2 do not recognize any nationality, boundaries, or political affiliation, but can nonetheless become important political issues (3). Therefore, managing the current crisis requires the highest political and diplomatic support in all countries. In this context, the Director-General has also expressed his concerns regarding the extent to which political commitments could match the level of such crises (1) and emphasized the necessity of global accountability toward these threats (4). In 2003, the SARS crisis provided an opportunity for countries to adopt international health regulations to reach a “cosmopolitan moment” to address the weaknesses in responding to its outbreak (3). However, the current COVID-19 pandemic has affected all the developing and developed nations and not like the SARS, a regional spread with effective containment. Considering the current bottlenecks in pandemic control, multilateralism, global solidarity, and strengthening international collaboration, it is crucial to ensure success (5). Global coordination and cooperation are critical during pandemics, and the current pandemic showed many signs of its failure and lack of proper leadership at the global level (6). However, a move away from health diplomacy with global involvement is likely to be marked by competition and a lack of coordination (7). In this paper, we discuss the scope of global health diplomacy (GHD) and how it can be applied and practiced for strengthening global arenas, such as International Cooperation, Global Solidarity, Global Economic Trade and Development, Global Health Security, Strengthening Health Systems, and addressing inequities and inequalities.

## Why Global Health Diplomacy Now and How Can Countries Benefit?

**Figure 1:**
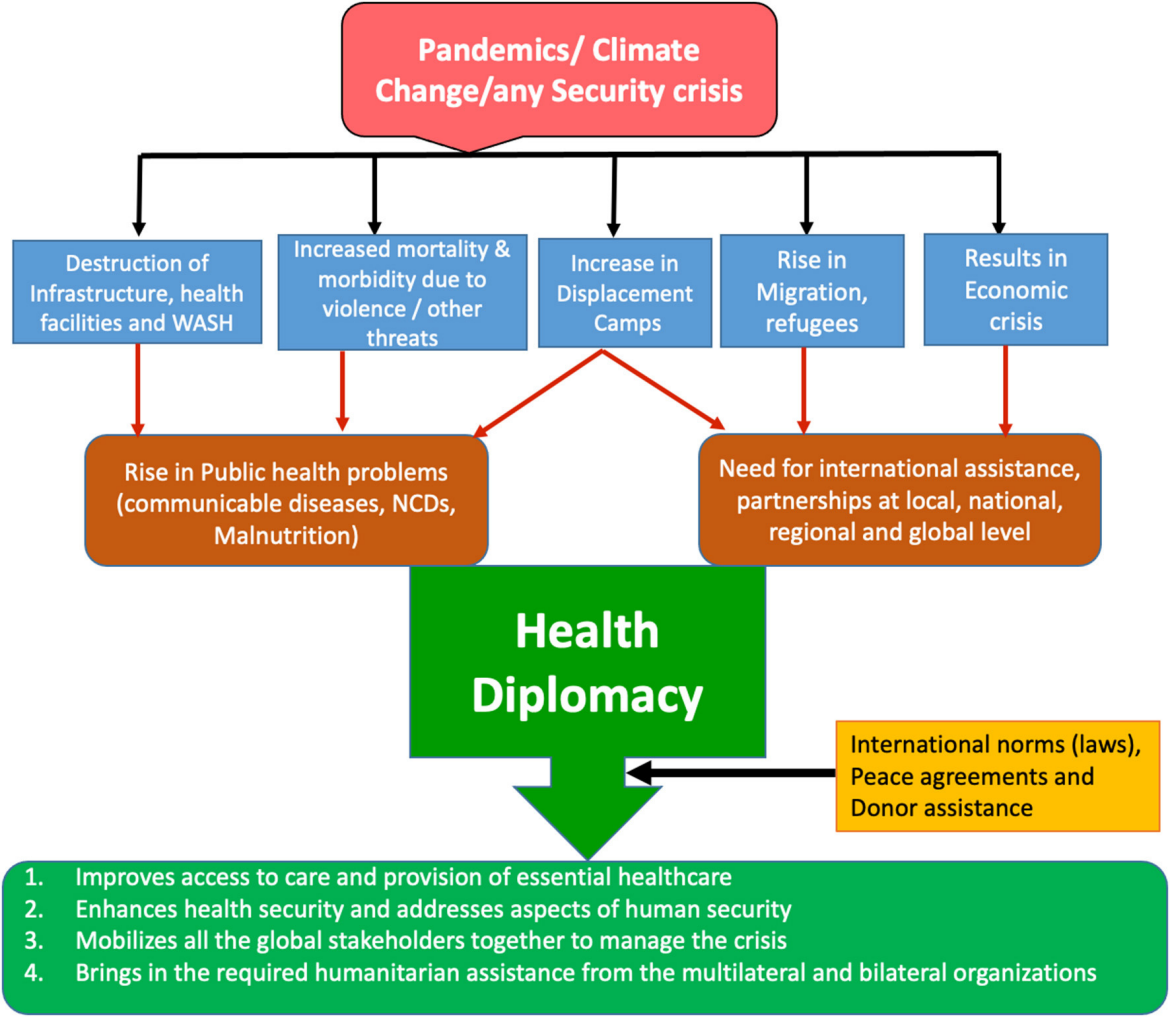
Addressing global health threats and crises through health diplomacy. Source: Prepared by the authors.

According to the WHO, GHD connects public health, law, international affairs, management, and economics, concentrating on negotiations and shaping the global policy climate for health. The central tenet of GHD is for countries to work together in international fora to address public health issues (8). According to Kickbusch, GHD refers to the multi-level and multi-actor negotiation processes that shape and manage the global policy environment for health in health and non-health fora (9). Moreover, the health problems/threats addressed diplomatically have also become diverse, ranging from neglected tropical diseases, growing antimicrobial resistance (AMR), infectious diseases, accessibility of medicines, sale of unsafe, counterfeit drugs, to brain drain crisis. Hence, global health has become more diverse as the actors widened and the interests appealed to the traditional humanitarian ideals associated with health and the principles grounded in national and global security (10). Currently, the world is facing many challenges such as the ongoing COVID-19 pandemic, climate change, increasing refugees and migration, growing rivalries among nations due to the geopolitical shifts. The whole scenario indicates that no country in any corner of the world is safe and secure, thereby indicating the importance of global solidarity as the health threats transcend the borders in no time in this globalized world. To address these issues peacefully and diplomatically, countries are also undergoing a digital transformation to get engaged in diplomacy through virtual/online summits as these complicated negotiations could not be conducted face-to-face at a point in time where it is more necessary than ever. Therefore, through GHD, more holistic, inclusive, comprehensive, and coordinated strategies could be worked out to address the global challenges (health and non-health related) by focusing on the SDGs addressing the partnerships, human development, and peace (11). A hypothetical scenario depicted below shows how a typical emergency situation in any nation or region can create complications and how these challenges can be addressed by multi-sectoral coordination and global governance frameworks through the successful practice of GHD based on the international norms and agreements (Figure 1).

Hence, GHD is very critical and successful in addressing many of the global challenges as we have witnessed in the formulation of International Health Regulations (IHRs 2005), Framework Convention of Tobacco Control (FCTC), Universal Health Care (UHC) (12), Sustainable Developmental Goals (SDGs), UN Climate Change Conference in December 2019 and most recently the COVAX Facility in 2020 to address the COVID-19 pandemic (13).

## Results

**Figure 2:**
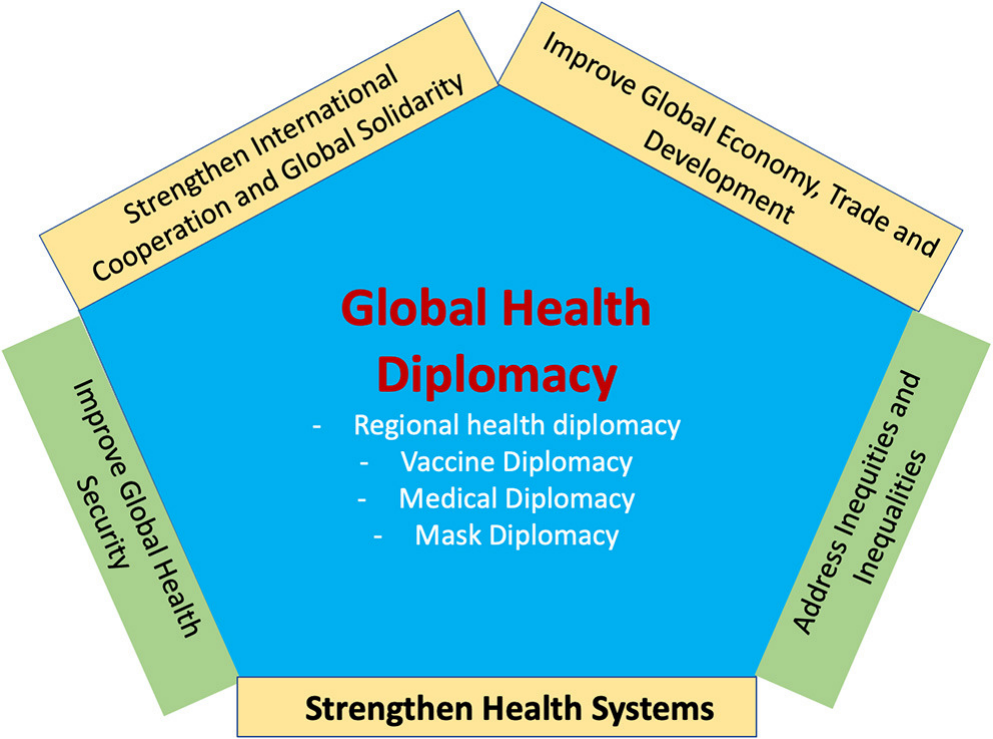
Global health diplomacy and the five key areas of impact.

This paper has highlighted the five main areas (Figure 2) where GHD could contribute and make significant progress in tackling the global challenges of developing and developed nations.

### International Cooperation and Global Solidarity Through GHD

Health is now a part of global food, environment, oil, and water agreements, and it is addressed at major global and regional summits, including the G7 and G20 summits. Every SDG that has been negotiated has shown that health is an important component and outcome. Therefore, this explains why GHD plays a central role in every subsequent round of SDG-related negotiations. Given this background, the global health agenda is now viewed as a common challenge for developing and developed nations (13). COVID-19 offers many different examples of health diplomacy but is mostly characterized by fragmentation. There is much evidence showing the aspects of poor leadership and cooperation between big countries. Amid this fragmentation, as a regional response, the European Union (EU) vaccine initiative emerged recently independent of the WHO (7). GHD can be interpreted as a political shift toward achieving the goals of improving global health while maintaining and strengthening broad international relations, particularly in the areas affected by conflicts and limitation of resources (14). The first UN General Assembly resolution on the coronavirus (UNGA 270.74) called for international cooperations to combat the virus (5). In short, we discuss a few large economies that failed to show their leadership or direction to the other developing nations in many aspects and have failed even in diplomacy with their counterparts.

In the case of previous epidemics of HIV, SARS, H1N1, MERS, Zika, and Ebola, they have mostly affected specific regions/nations or the global south more than the north, and therefore the support came in from the rich nations in Western Europe and North America. However, in the current COVID-19 pandemic, the rich nations have been affected more, with more cases and deaths resulting in global competition, lack of solidarity, and nationalist movements in addressing the domestic economic and health crises (15).

In the United States' federalist system of public health governance, COVID-19, which is highly transmissible, crosses the borders efficiently, and impacts the economy and infrastructure, have exposed many weaknesses. Besides, the federal response was alarmingly slow to evolve, causing uncertainty about the existence of the virus and the measures taken to tackle it (16). Despite being the wealthiest with the most advanced systems, the US had the highest burden of infections and deaths globally. As of May 17, 2021, there were 32,605,236 confirmed cases and 580,166 deaths (2). The ill-timed decision of the President of the United States of America to stop supporting the WHO, that the rise of populist sentiment worldwide, is another blow to the multilateral structure that reflects a nationalist agenda (17). Therefore, it is worth noting the significant role of GHD strategies in addressing the friction between populism and multilateralism in this context. For example, the WHO incorporates and engages in GHD with its strategic alliance partners to balance and address the challenges posed by the populist agendas to address the critical aspects (equity, accessibility, affordability, and availability of services) for the needy and undeveloped regions/nations.

In China, following the outbreak of COVID-19 in Hubei province, China, the government imposed a lockdown on its cities. Still, the international air travel through flights was not canceled, which resulted in people traveling from outside to China and vice versa. Though China was initially seen primarily as the source of the virus, it has started to provide material and equipment such as masks, PPE to its neighbors, the Middle East, and Europe (18). These actions display its engagement in soft power and change its image in front of the global community (19). Since the states are the key actors that make binding agreements through the extension of their interests through diplomatic practices (Core health diplomacy), they are, by default, part of the GHD. However, there were also complaints against China for sending the poor-quality masks, and test kits supplied to various countries (20). During this pandemic, the rivalry between the US and China has increased due to the conspiracies about the source of the virus, which has presented a substantial challenge to GHD and even more challenging is a lack of global leadership (7).

With 15.58 million confirmed cases and 434,715 deaths as of May 17, 2021 (2), Brazil is among the top three countries with high mortality, evidence of underreporting (21), and high death rates among health professionals, pregnant women (22), and the indigenous population. Ferigato et al. pointed out that while there is a great need for a concerted political response driven by social justice and evidence-based expertise while handling any public health crises such as COVID-19 with enormous economic and health impacts, this is sadly not happening in Brazil (23).

The impact of the COVID-19 pandemic, the withdrawal of the funding to the WHO by the US President, has resulted in an unprecedented situation. This, in turn, has affected the health of people living in the Americas as the Pan American Health Organization (PAHO) being a beneficiary (24) and the fragile economies, reducing the ability of health organizations to help manage endemic pandemic, and neglected tropical diseases (25). To emphasize, WHO and its six regional offices played a vital role during this pandemic, which should be commended. The WHO regional offices made major contributions to countries through cooperation, technical assistance, development of financial mechanisms through global partnerships, implementation of COVID-19 standards & case management guidelines, provision of essential deliverables (vaccines, vital medicines, PPEs, and testing kits), and infodemic management to guide everyone. Through GHD, the countries can help each other by assisting wherever possible and by supplying the essential personal protective equipment (masks, gloves, disposable gowns, disinfectants, etc.), essential drugs, health care resources, working together for new diagnostics, vaccines, etc. as done by some nations through “mask diplomacy”- by sending the face-masks, e.g., China, Taiwan; medical diplomacy- through doctors/ health care staff, and vaccine diplomacy, e.g., India by sending vaccines to other nations to gain the goodwill. This COVID-19 pandemic has the potential to galvanize the long-needed global cooperation. There is an immediate need for cooperation, collaboration, mutual responsibility, and critical traits such as transparency, accountability, trust, and fairness (26). As emphasized by Hoffman, effective global governance is not possible when countries cannot depend on each other to comply with international agreements (27).

### Improving Global Economy, Trade, and Development Through GHD

The rapid spread of the current epidemic taught us that the international environment could not be managed as an island. International solidarity and convergence and the abandonment of political games could be part of a related approach to confront neo-isolationism. However, isolationism (as long as not coupled with xenophobia) can also have benign effects from a health security perspective regarding the movement for disease vectors. Therefore, the more the global economy is affected, the greater political priority is paid to health and health diplomacy (28).

Perhaps political leaders apply GHD as a soft tool; instead of taking impulsive actions, the fear of the global community of the spread of coronavirus could have been curbed (14). The declaration in Oslo of the ministers, a sign of the growing role of global health in foreign policy, calls on governments to move toward a diplomatic approach that addresses public health concerns. However, non-compliance to these ideas and neo-nationalism (particularly) may have resulted in serious problems in the way diplomats seek to preserve strategic relations with their allies (29). Although international trade cooperation has suffered geopolitical rivalry and shifts, governments can strengthen the nexus between public health and trade policies through GHD to fight the pandemic (26).

In many recent cases, countries' officials initially denied, downplayed its severity, and even adopted a blame game strategy. The latter was represented by some leaders pointing the finger of blame at other countries for the spread of the disease and trying to bypass and downgrade the role of the WHO as the central coordinator in controlling the epidemic. The non-compliance of some countries with international health regulations (IHRs) and poor international and regional cooperation has been documented (30). Moreover, two-thirds of these countries have not reported their additional health measures to WHO (ref), which is a further violation of IHR articles 43.3 and 43.5 as highlighted by Habibi et al. in a recent Lancet article (31). The effectiveness of decision-making at WHO depends on Member States' political will to find a common solution. Fundamental disagreements between the Member States, especially between powerful ones, can block political decision-making at WHO. However, the Organization can often preserve its normative function (that is, the setting of technical norms and standards) (13). The agency does not have the authority to investigate epidemics within countries independently, and as mentioned above, it has no enforcement power. The current crisis also reminded humanity that even the most powerful governments of the world and the wealthiest people are helpless in the face of the negative consequences of this disease (32). It once again highlighted our macro and micro-level interdependencies and drew the attention of the world toward strengthening GHD and international collaborations as crucial tools to cope with an emerging challenge as not an option but a necessity. All countries should prioritize and find common resource allocation, common interests, and common operational overlap on development issues.

The Trade-Related Aspects of Intellectual Property Rights (TRIPS Agreement) and Public Health as part of the Doha Declaration has stated key flexibilities to countries in Article 31. Furthermore, clause 5(c) of the TRIPS Agreement stated that “public health crises, including those relating to HIV/AIDS, tuberculosis, malaria and other epidemics,” can constitute “a national emergency or other circumstances of extreme urgency.” The clause provides countries with some flexibility in managing the patents for pharmaceuticals (public goods), especially in situations of “national emergencies” and “other circumstances of extreme urgency” (33–35). However, in October 2020, India and South Africa have jointly put up a drafted proposal for a temporary waiver for the Intellectual Property Rights (IPRs) in World Trade Organization (WTO) to address the affordability and accessibility of the COVID-19 drugs to one and all. However, this proposal was rejected by nine WTO members, including the European Union, through 100 countries showed support for the proposal (36). In this context, Chattu et al. emphasize that the IPRs regime should not become a barrier to accessibility/affordability of essential drugs and vaccines for COVID-19. To succeed and ensure access, India and South Africa need to get more engaged in GHD with all the involved global stakeholders to get strong support for their joint proposal (15). If the TRIPS waiver request is accepted, access to vital COVID-19 drugs, technologies, and diagnostics can be greatly improved. As a result, there is a great need for involving physicians trained in GHD in shaping global trade policies and being the undisputed authorities in the realm of health to counterbalance the overwhelming influence of multi-national companies/corporations with their corporate interests and profit-seeking agenda (37). Amid this crisis, there are opportunities to help all countries improve their international public health landscape position by adopting appropriate strategies and intelligent foresight and forecasting.

### Improving Global Health Security Through GHD

The Global Health Security Initiative (GHSI) is an international partnership to strengthen health preparedness and response globally to biological, chemical, radio-nuclear, and pandemic influenza threats. They were launched in November 2001 by Canada, the European Commission, France, Germany, Italy, Japan, Mexico, the United Kingdom, and the United States of America. WHO provides technical support to the initiative, which is again a result of successful GHD (38). The global health policy of WHO is focused on three pillars: Universal Health Coverage (UHC), health crises, and improved health and well-being (39). In the context of the COVID-19 syndemic, it would be easy to focus attention on global health security by improving public health and healthcare systems. Health security is an environment that is intrinsically political and sensitive. The prospect of harm to relations between the developed and developing world is threatened by any new bilateral or multilateral policies or initatives (40). A recent Lancet editorial highlights COVID-19 as a Syndemic of coronavirus infection combined with an epidemic of NCDs; both interact on a social substrate of poverty and inequality. The Global Burden of Disease (GBD) research group emphasizes that unless structural inequities and inequalities deeply embedded in society are addressed, a more liberal approach to immigration policies is adopted. Therefore, population health will not achieve the benefits that global health advocates seek. It is time to shift course for the global health community (39). GHD is viewed as a compulsory tool in smart diplomacy, especially during the emergencies such as outbreaks, epidemics, and pandemics, for a timely and effective response from the global community. As highlighted by Javed et al., a lack of clear communication and coordination among the WHO member states regarding travel restrictions and bans indicated a violation of IHRs (26). A decade ago, the IHRs were introduced shortly after the extreme acute respiratory syndrome (SARS) to speed up international cooperation in public health emergencies. While these regulations are legally binding, they have some shortcomings, such as the lack of a compliance framework. Any public health incident that may constitute a Public Health Emergency of International Concern (PHEIC) must be notified to WHO by all governments. PHEIC has already been used to combat the global spread of infectious diseases such as Polio, H1N1, Ebola, Zika (41), and most recently, COVID-19.

The health diplomacy landscape is essential if and when the multifaceted and devastating consequences of this hidden enemy subside (42). It is expected to be the central component of regional and global networks for collaborations to combat epidemics (42), strengthening multilateralism (39) and international cooperation in global issues (28). It could also act as a soft power to achieve the geopolitical influence (3, 42) deescalate political tensions between and among nations. The practice of health diplomacy also enhances the cohesion of national, regional, and continental efforts to resolve health crises (42), attain a political reputation in the international arena, and be recognized as a global health benevolant (3) in reinforcing international peace and stability (43).

Health has become an issue of national security/global concern, and GHD aids in developing new bilateral or multilateral agreements to safeguard the health and well-being of people. As a result, many countries have implemented the IHRs, imposed travel restrictions, and implemented public health regulations. Some countries that enacted them in the early days of the pandemic are Canada (44), India (45), and the Caribbean region (46), to name a few. The extensive consequences of the COVID-19 pandemic at various levels are not yet clear, but it could certainly prove to be a prelude to closer cooperation at the national, regional, and global levels. No phenomenon is a pure threat; depending on how we respond, it also could bring opportunities. World leaders are now expected to revise foreign policies toward health diplomacy, as taking advantage of the hidden opportunities of this crisis requires a flexible and innovative foreign policy. They should also be aware that short-sighted decisions and policies that weaken the health system and intensify the limitations of financial resources available for managing the current epidemic not only is a threat to the health of their nation but will also be just as threatening to the health of the people of the world. Today, most national health policies are international, considering the current growing interdependencies among countries.

### Strengthening Health Systems Through GHD

Once again, the pandemic has proved that health should be placed at the center of the international agenda and considered the primary concern of countries' foreign policy strategies (29). The key components of any robust public health and healthcare sector are qualified and robust health workers; safer, more efficient, and high-quality service delivery; health information systems; access to essential medicines; adequate funding; and good governance. The global shortage of personal protective equipment (PPE) for frontline health care staff, such as surgical masks, N95 masks, respirators, hand sanitizers, gloves, face shields, disposable gowns, and other products, posed an ethical challenge as well as a major barrier to pandemic preparedness (26). Moreover, the COVID-19 pandemic has crippled many health systems due to its double impact as a syndemic where people worldwide are also experiencing the epidemic of NCDs concurrently.

In recent years, health has been something more than just a “purely humanitarian” effort based on national interests in helping to achieve other functions of foreign policy (47). We should again perhaps go beyond the policing views, security lenses, and human rights grounds for intervention; preserving human dignity should be instead at the center of all adopted strategies and responses. It is now more than ever necessary for governments to be politically committed to achieving “health for all” and promoting the “One Health” approach to reduce the risk of future infectious diseases and improve the global response to pandemics. However, during the COVID-19 pandemic, though the health diplomacy funneled through WHO has been fraught, the developing countries have received assistance from the World Bank and the International Monetary Fund. Many global and multilateral organizations' efforts have stalled, and, in this connection, even the G20 have put statements emphasizing international commitment. Still, most of the measures are limited to domestic fiscal policy (48).

What COVID-19 pandemic has interestingly and ironically exposed to the world is such that many European and North American countries—arguably some of the best health systems of the world—are crumbling and struggling to contain the widespread infections; while some of the developing countries' health systems, as well as smaller health systems in Asia (notably those in Asia such as Thailand, Vietnam, Taiwan, Singapore, and China), are doing much better, evidenced by the lower number of infections and lower fatality rates in these health systems. Even though the Western European and North American health systems have higher capacities and are equipped with hi-technology equipment, they all suffered a severe strain during this pandemic. Nonetheless, one can also argue that it is the initial political and policy responses adopted by different countries toward the pandemic that put them on different trajectories. The initial policy directions had a huge influence and impact on health system capacities in the context of the pandemic as witnessed by the immediate actions by the heads of the states in Asia (as mentioned above) along with Canada (44), India (45), and Caribbean states (46) to impose the travel bans and public health regulations.

The recommendations of the Global Preparedness Monitoring Board for pandemics and emergency public health situations requires strengthening multilateral collaborations and closer cooperation between governments and other actors of the international system as well as mutual trust (28): (1) full compliance with the international health regulations; (2) legal obligations of countries to combat the spread of disease; (3) surveillance and responding to possible pandemics to avoid their becoming a serious risk; (4) supporting the poorest countries to prepare for global threats. Moreover, collaboration in establishing formal and confidential channels for free sharing and transmission of information on possible health threats could have prevented the stigma attached to those countries where the outbreak originated (29). Also, for the developing nations, especially for the African countries, it was emphasized that it would be prudent for African CDC to embrace GHD to strengthen their capacities for disease preparedness and response (49).

### Addressing Equity and Health-Related Global Targets

If well-conducted, GHD results in improved global health, greater equity, better relations and trust between states, and a strengthened commitment on the part of stakeholders to work together with stakeholders nationally and globally (13). Many countries in conflict and war face unique problems such as a serious humanitarian crisis that need to work even harder to deal with the consequences of the pandemic. Some of the challenges include inaccessibility to health care services, lack of medical services, halting of research collaborations, and the “chilling effect” due to the withdrawal of humanitarian and health staff from these countries. These dangers can destabilize conflict-ridden countries and have devastating and long-term consequences for countries in the neighboring regions (26). GHD ensures that governments are serious about health-related results, prioritizing context-specific health needs in securing bilateral/multilateral support for addressing crucial macro-level, non-medical initiatives (outside of national health systems) to achieve the goals of Universal Health Coverage (UHC) and health-related SDGs (50).

The primary goal of health diplomacy should be to reduce inequalities by making diagnostics, therapeutics, and vaccinations a global public good accessible to all. GHD will serve as a bridge for international collaboration in addressing public health issues, improving health services, and rebuilding multilateral institutions by emphasizing universal health coverage for sustainable and equitable growth (26). The aspects of access and affordability of healthcare services are emphasized through the UHC targets and the health-related sustainable development goals. There is a need to recognize and reemphasize that health can offer a good entry point for dialogue to promote peace and global health security through GHD (51). Moreover, the sole purpose of the health domain is for serving the public interests, and therefore it can be used as a medium in building trust and legitimacy through the practice of GHD with an ultimate goal of achieving the SDGs aimed at human development (26). International organizations such as the World Bank, WHO, along with the Bill and Melinda Gates Foundation and other International NGOs, have raised a fund of US$ 8.1 billion and introduced the WHO COVAX plan for the fair and equitable distribution of an eventually licensed vaccine (52). The COVAX Initiative is an instrument for a fairer global distribution resulting from successful GHD, but the end result can only be assessed after some time based on fulfilling its commitments to the nations. Currently, there are many challenges with the second wave of COVID-19 pandemic in India, resulting in an acute shortage of vaccines, and the Serum Institute, which had received the contract to manufacture the vaccines under COVAX facility domestic demand and international shipments. As per the latest report, COVAX has a COVID-19 vaccine shortage of 190 million doses, and the few manufacturers that have signed agreements with the facility can only supply later this year or only in 2022 (53). The report of WHO on Health in All Policies “(HiAP) in 2010 aims to collaborate” across sectors to achieve common goals. HiAP is a strategy to include health considerations in policy making across different sectors that influence health, such as transportation, agriculture, land use, housing, public safety, and education. HiAP reaffirms the essential role of public health in addressing policy and structural factors affecting health (54). Though HiAP is not implemented in most countries leading to weaker and fragile health systems with many inequities and inequalities, the role of health diplomacy is very critical to fill these gaps and implement these effective policies through international cooperation and successful negotiations for revamping the infrastructure, technology and disease surveillance in many low -and middle- income countries (26). AlKhaldi et al. GHD emphasized that science diplomacy, vaccine diplomacy, and equitable scientific cooperation are new powerful instruments that must be used to unite the world and create a safer and more integrated society (55). Therefore, UHC and SDGs can only be accomplished if the underlying root causes, such as social, gender, and health inequities, are addressed accordingly.

## Conclusions

The pandemic has uncovered many weaknesses of the global health systems and increased the burden on the already fragile healthcare systems in many countries. The world has witnessed many nationalistic movements in many countries to secure drugs, vaccines, and personal protective equipment with a selfish attitude, which is not the right choice. The virus has no boundaries, and unless the nations work together with solidarity and help the developing countries with fragile health systems to build their health systems, health security cannot be ensured. Countries must improve their health systems, focusing on surveillance systems, infrastructure and investing more in science and technology to solve the challenges of today and in the future in more scientific and technologically sound ways. The GHD plays a critical role as the nations have been sensitized about the importance of the health sector and their citizens' health, ultimately running the nations' economies. It is high time that the Science, Technology, and Health domains should work more closely and contribute to the development of novel technologies, vaccines, drugs, medical devices, and kits for COVID-19 and future health threats. The countries should also prioritize and create expert teams in GHD at the national level to frame their interests and negotiate at the global platforms for better outcomes. Through successful GHD, the health ministries and the global health professionals could frame health as a high priority and reemphasize the strategy of Health in All Policies (HiAP) for a safer post-pandemic world. GHD can strengthen international cooperation, health systems, improve the global economy, trade, and address the inequities to achieve health-related global targets. Therefore, GHD can deal with several complex issues in the multipolar world which have strongly linked geo-socio-economic and political determinants and pave the way for health, development, security, and peace.

## Data Availability Statement

The original contributions presented in the study are included in the article/supplementary material, further inquiries can be directed to the corresponding author/s.

## Author Contributions

ST, VC, EJ, and SK: conceptualization and preparation of initial draft. VC and ST: literature review. VC: data collection and analysis, preparation of figures, editing, and finalizing the manuscript. All authors have approved the final version of the draft before submission.

## Conflict of Interest

The authors declare that the research was conducted in the absence of any commercial or financial relationships that could be construed as a potential conflict of interest.

## References

[1] Raoofi A Takian A Olyaeemanesh A Haghighi H Aarabi M. COVID-19 pandemic and comparative health policy learning in Iran. Arch Iran Med. (2020) 23:220–34. 10.34172/aim.2020.02 32271594

[2] World Health Organization. WHO Coronavirus Disease (COVID-19) Dashboard. Available online at: https://covid19.who.int/?gclid=CjwKCAjwq832BRA5EiwACvCWsV3mKW7qHOeakPWdLDSEiGEtQOe_7Q4o4XLlVR8bciBxvrVhP3kG99xoCBvUQAvD_BwE (accessed March 28, 2021).

[3] Kickbusch I Leung GM Bhutta ZA Matsoso MP Ihekweazu C Abbasi K. COVID-19: how a virus is turning the world upside down. BMJ. (2020) 369:m1336. 10.1136/bmj.m1336 32245802

[4] Zandifar A Badrfam R. Fighting COVID-19 in Iran; economic challenges ahead. Arch Iran Med. (2020) 23:284. 10.34172/aim.2020.14 32271606

[5] COVID-19 - Joint Declaration of the Alliance for Multilateralism. (2020). Available online at: https://www.diplomatie.gouv.fr/en/french-foreign-policy/united-nations/alliance-for-multilateralism-63158/article/covid-19-joint-declaration-of-the-alliance-for-multilateralism-16-apr-2020 (accessed May 31, 2020).

[6] Javed S Chattu VK. Ineffective COVID-19 pandemic response due to failed global leadership and international cooperation? Reimagining the future through effective global health diplomacy. Health Promot. (2020) 10:2. 10.34172/hpp.2020.45 PMC7723006 33312925

[7] Fazal TM. Health Diplomacy in Pandemical Times. International Organization (2020). p. 1–20.

[8] WHO. Policy Brief Health Diplomacy. Regional Office for the Eastern Mediterranean, World Health Organization (2014). Available online at: https://applications.emro.who.int/docs/Policy_Brief_2014_EN_15340.pdf?ua=1&ua=1&ua=1&ua=1&ua=1&ua=1 (accessed March 23, 2021).

[9] Kickbusch I Silberschmidt G Buss P. Global health diplomacy: the need for new perspectives, strategic approaches and skills in global health. Bull. World Health Organ. (2007) 85:230–2. 10.2471/BLT.06.039222 17486216 PMC2636243

[10] Chattu VK. The rise of global health diplomacy: an interdisciplinary concept linking health and international relations. Indian J Public Health. (2017) 61:134–6. 10.4103/ijph.IJPH_67_16 28721965

[11] Chattu VK Aslanyan G. Global health partnerships and translation. In: Haring R Kickbusch I Ganten D Moeti M editors. Handbook of Global Health. Cham: Springer (2021). p. 1–33.

[12] United Nations. Political Declaration of the High-Level Meeting on Universal Health Coverage: Moving Together to Build a Healthier World. (2019). Available online at: https://www.un.org/pga/73/wp-content/uploads/sites/53/2019/07/FINAL-draft-UHC-Political-Declaration.pdf (accessed August 15, 2020).

[13] Kickbusch I Nikogosian H Kazatchkine M Kökény M. A Guide to Global Health Diplomacy. Graduate Institute of International and Development Studies, Global Health Centre (2021). Available online at: https://www.graduateinstitute.ch/sites/internet/files/2021-02/GHC-Guide.pdf (accessed March 25, 2021).

[14] Adams V Novotny TE Leslie H. Global health diplomacy. Med Anthropol. (2008) 27:315–23. 10.1080/01459740802427067 18958783

[15] Chattu VK Singh B Kaur J Jakovljevic M. COVID-19 vaccine, TRIPS and global health diplomacy: India's role at the WTO platform. Biomed Res Int. (2021) 1–9. 10.1155/2021/6658070 PMC8416375 34485525

[16] Haffajee RL Mello MM. Thinking globally, acting locally—the US response to COVID-19. N Engl J Med. (2020) 382:e75. 10.1056/NEJMp2006740 32240580

[17] Trump Stops U.S. Funding of W.H.O.; U.K. Coronavirus Deaths May Be Higher Than Official Toll. The New York Times (2020). Available online at: https://www.nytimescom/2020/04/14/world/coronavirus-news-world-global-live.html. (accessed October 24, 2020).

[18] Burton G. China COVID-19 in MENA. POMEPS (2020). Available online at: https://pomeps.org/china-and-covid-19-in-mena (accessed August 15, 2020).

[19] Joseph Nye “Soft Power ” Foreign Policy. Joseph Nye, Soft Power: The Means to Success in World Politics. New York, NY: Public Affairs (2004). p. 153–71.

[20] Soylu R. Coronavirus: Turkey Rejects Chinese Testing Kits Over Inaccurate Results. Middle East Eye (2020). Available online at: https://www.middleeasteye.net/news/coronavirus-turkey-faulty-chinese-kits-not-use (accessed March 31, 2020).

[21] Moraes T Barberia L. COVID-19: Public Policies and Society's Responses. Quality Information for Refining Public Policies and Saving Lives. Policy Briefing Note 20. Rede de Pesquisa Solidária de Políticas Públicas e Sociedade, São Paulo (2020).

[22] Takemoto ML Menezes MD Andreucci CB Nakamura-Pereira M Amorim MM Katz L. The tragedy of COVID-19 in Brazil: 124 maternal deaths and counting. Int J Gynecol Obstetr. (2020) 151:154–6. 10.1002/ijgo.13300 32644220 PMC9087660

[23] Ferigato S Fernandez M Amorim M Ambrogi I Fernandes LM Pacheco R. The Brazilian Government's mistakes in responding to the COVID-19 pandemic. Lancet. (2020) 396:1636. 10.1016/S0140-6736(20)32164-4 33096042 PMC7575269

[24] King A Andrus JK Figueroa JP. Financial crisis at PAHO in the time of COVID-19: a call for action. Lancet. (2020) 396:96. 10.1016/S0140-6736(20)31489-6 32622372 PMC7332277

[25] Baranchuk A Liprandi AS Wyss F Pineiro D. Ending support for medical organizations puts the world at risk. Lancet. (2020) 396:1398. 10.1016/S0140-6736(20)32155-3 33091359 PMC7572100

[26] Javed S Chattu VK. Strengthening the COVID-19 pandemic response, global leadership, and international cooperation through global health diplomacy. Health Promot Perspect. (2020) 10:300. 33312925 10.34172/hpp.2020.48 PMC7723006

[27] Hoffman SJ. The evolution, etiology and eventualities of the global health security regime. Health Policy Plann. (2010) 25:510–22. 10.1093/heapol/czq037 20732860

[28] Momtazmanesh S Ochs HD Uddin LQ Perc M Routes JM Vieira DN. All together to fight COVID-19. Am J Trop Med Hyg. (2020) 102:1181–3. 10.4269/ajtmh.20-0281 32323644 PMC7253116

[29] Nabi J. The Case for Global Health Diplomacy. (2020). Available online at: https://www.project-syndicate.org/commentary/coronavirus-global-health-diplomacy-three-strategies-by-junaid-nabi-2020-02 (accessed May 31, 2020).

[30] World Health Organization. Novel Coronavirus (2019-nCoV): Situation Report-18. Available online at: https://www.who.int/docs/default-source/coronaviruse/situation-reports/20200207-sitrep-18-ncov.pdf?sfvrsn=fa644293_2 (accessed May 16, 2021).

[31] Habibi R Burci GL de Campos TC Chirwa D Cinà M Dagron S. Do not violate the International Health Regulations during the COVID-19 outbreak. Lancet. (2020) 395:664–6. 10.1016/S0140-6736(20)30373-1 32061311 PMC7133591

[32] Islam S. Fighting Covid-19: International Cooperation is Needed. (2020). Available online at: https://moderndiplomacy.eu/2020/04/20/fighting-covid-19-international-cooperation-is-needed/ (accessed May 31, 2020).

[33] WTO. Ministerial Conferences - Doha 4th Ministerial - TRIPS Declaration. WTO (2001). Available online at: https://www.wto.org/english/thewto_e/minist_e/min01_e/mindecl_trips_e.htm (accessed July 31, 2020).

[34] WTO. Intellectual Property (TRIPS) - Fact Sheet: TRIPS and Pharmaceutical Patents. WTO (2006). Available online at: https://www.wto.org/english/tratop_e/trips_e/factsheet_pharm02_e.htm (accessed July 31, 2020).

[35] UNDP. Using TRIPS Flexibilities to Improve Access to HIV Treatment. United Nations Development Programme (2015). Available online at: https://www.undp.org/content/undp/en/home/librarypage/hiv-aids/using_trips_flexibilitiestoimproveaccesstohivtreatment.html (accessed March 25, 2021).

[36] WTO. (IP/C/W/669). Waiver From Certain Provisions of the TRIPS Agreement for the Prevention, Containment and TREATMENT of COVID-19. (2020). Available online at: https://docs.wto.org/dol2fe/Pages/SS/directdoc.aspx?filename=q:/IP/C/W669.pdf&Open=True (accessed December 30, 2020).

[37] Chattu VK Pooransingh S Allahverdipour H. Global health diplomacy at the intersection of trade and health during the COVID-19 era. Health Promot Perspect. (2021) 11:1–4. 10.34172/hpp.2021.01 33758749 PMC7967129

[38] Global Health Security Agenda. Available online at: http://www.ghsi.ca/english/index.asp (accessed March 24, 2021).

[39] Florent V. Global health: time for radical change?. Lancet. (2020) 396:1129. 10.1016/S0140-6736(20)32131-0 33069323 PMC7561297

[40] Chattu VK Kevany S. The need for health diplomacy in health security operations. Health Promot Perspect. (2019) 9:161. 10.15171/hpp.2019.23 31508335 PMC6717919

[41] Maurice J. Expert panel slams WHO's poor showing against Ebola. Lancet. (2015) 386:e1. 10.1016/S0140-6736(15)61253-3 26183412

[42] Sumaworo M. Medical Diplomacy to Face the Global Health Challenges: A Case Study of COVID 19. Available online at: https://frontpageafricaonline.com/opinion/commentary/medical-diplomacy-to-face-the-global-health-challenges-a-case-study-of-covid-19/Mar23. (2020) (accessed May 31, 2020).

[43] Chattu VK Knight A Reddy KS Aginam O. Global health diplomacy fingerprints on human security. Int J Prev Med. (2019) 10:2. 10.4103/ijpvm.IJPVM_11_19 31879553 PMC6921280

[44] Chattu VK Adisesh A Yaya S. Canada's role in strengthening global health security during the COVID-19 pandemic. Global Health Res Policy. (2020) 5:1–3. 10.1186/s41256-020-00146-3 32328533 PMC7167363

[45] Gauttam P Patel N Singh B Kaur J Chattu VK Jakovljevic. Public health policy of India and COVID-19: diagnosis and prognosis of the combating response. Sustainability. (2021) 13:3415. 10.3390/su13063415

[46] Chattu VK Chami G. Global health diplomacy amid the COVID-19 pandemic: a strategic opportunity for improving health, peace, and well-being in the CARICOM Region—a systematic review. Soc Sci. (2020) 9:88. 10.3390/socsci9050088

[47] Fidler DP Drager N. Health and foreign policy. Bull World Health Organ. (2006) 84:687. 10.2471/BLT.06-035469 17128330 PMC2627479

[48] Jain A. Trump Just Missed a Perfect Opportunity to Reassert American Leadership. Foreign Policy (2020). Available online at: https://foreignpolicy.com/2020/04/02/g20-helped-beat-ebola-but-not-coronavirus/ (accessed July 24, 2020).

[49] Chattu VK Pooransingh S. Strengthening African health systems through global health diplomacy. Health Promot Perspect. (2020) 10:292. 33312922 10.34172/hpp.2020.45 PMC7723004

[50] Chattu VK Knight WA Adisesh A Yaya S Reddy KS Di Ruggiero E. Politics of disease control in Africa and the critical role of global health diplomacy: a systematic review. Health Promot Perspect. (2021) 11:20–31. 10.34172/hpp.2021.04 33758752 PMC7967135

[51] Chattu VK Knight WA. Global health diplomacy as a tool of peace. Peace Rev. (2019) 31:148–57. 10.1080/10402659.2019.1667563

[52] von Bogdandy A Villarreal P. The Role of International Law in Vaccinating Against COVID-19: Appraising the COVAX Initiative. Max Planck Institute for Comparative Public Law & International Law (MPIL) Research Paper (2020).

[53] Cullinan K. WHO Appeals For Vaccine Donations to Cover Huge COVAX Shortfall. (2021). Available online at: https://healthpolicy-watch.news/who-appeals-for-dose-donations-to-cover-huge-covax-shortfall/ (accessed May 17, 2021).

[54] Kickbusch I. Health in all policies: the evolution of the concept of horizontal health governance. In: Kickbusch I Buckett K editors. Implementing Health in All Policies. Adelaide, SA: Rundale Mall: Government of South Australia (2010). p. 11–24.

[55] AlKhaldi M James N Chattu VK Ahmed S Meghari H Kaiser K. Rethinking and strengthening the Global Health Diplomacy through triangulated nexus between policy makers, scientists and the community in light of COVID-19 global crisis. Global Health Res Policy. (2021) 6:1–6. 10.1186/s41256-021-00195-2 33845923 PMC8041471

